# Comment on Matsuo et al. Impact of Olfactory Change on Postoperative Body Weight Loss in Patients with Gastric Cancer after Gastrectomy. *Nutrients* 2024, *16*, 851

**DOI:** 10.3390/nu16152422

**Published:** 2024-07-26

**Authors:** Sang Yeoup Lee

**Affiliations:** 1 Family Medicine Clinic, Biomedical Research Institute, Pusan National University Yangsan Hospital, Yangsan 50612, Republic of Korea; saylee@pnu.edu; Tel.: +82-55-3601442; 2 Integrated Research Institute for Natural Ingredients and Functional Foods, Yangsan 50612, Republic of Korea; 3 Department of Medical Education, Pusan National University School of Medicine, Yangsan 50612, Republic of Korea

I read with interest the paper by Matsuo et al. [1] entitled “Impact of Olfactory Change on Postoperative Body Weight Loss in Patients with Gastric Cancer after Gastrectomy”. The paper reported that 17.2% of patients following gastrectomy for gastric cancer experienced post-surgical olfactory alterations. Notably, it emphasized that the cohort with these olfactory changes exhibited significantly higher rates of postoperative weight loss than individuals whose olfactory function remained intact. A 2003 retrospective study by Harris and Griffin [2] identified taste and/or smell deficits following surgery in patients with upper gastrointestinal cancers. However, most research has primarily focused on the association between olfactory changes and metabolic surgery for obesity. This prospective study is the first to investigate the relationship between surgery and olfactory changes in patients with gastric cancers. The findings notably reveal that olfactory changes constitute an independent risk factor for weight loss one-month post-surgery. In daily clinical practice, this finding suggests that adding a routine evaluation of olfactory function to postoperative dietary counseling could significantly benefit patients with gastric cancer. However, I would like to make a few comments about the study.

The recent literature has reported that pylorus-preserving gastrectomy (PPG) was associated with reduced weight loss and better maintenance of nutritional status compared to other surgical procedures [3]. Interestingly, the current study observed a notably high prevalence (40%) of olfactory changes in the PPG group. The authors suggest a potential link between postoperative olfactory changes and significant weight loss [1]. Given this possibility, especially in the PPG group where olfactory changes were prevalent, it is essential to present detailed weight loss data for each group. Such data would further strengthen the suggested association.

The mechanisms of post-gastrectomy olfactory changes remain not fully understood. However, in addition to the potential role of the vagal pathway [4], leptin, and gastrointestinal symptoms in these changes [1], recent research on the relationship between ghrelin and olfactory changes is promising. In a recent study utilizing functional magnetic resonance imaging, ghrelin does not alter basic odor perception directly, such as the ability to detect or distinguish different odors. However, it has been shown to promote food odor conditioning, enabling individuals to respond faster and find food odor-associated cues more attractive [5]. 

Initially, weight loss following bariatric surgery was attributed mainly to reduced stomach capacity and malabsorption. However, subsequent research has revealed a more significant role in changes in gut hormones, leading to the current term bariatric metabolic surgery. A prominent hormonal change is a decrease in ghrelin, a hormone known to stimulate appetite. The stomach, especially the proximal fundus, is the main production area responsible for 70% of circulating ghrelin [6]. These findings regarding ghrelin and weight loss may also be applicable to stomach cancer surgery. Total or proximal gastrectomy in patients with gastric cancer demonstrates a large postoperative decrease in ghrelin and greater weight loss [7]. Olfactory changes are also common after bariatric metabolic surgery. Zerrweck et al. [8] reported a higher percentage of excess weight loss, 74% at 10 ± 6.7 months following laparoscopic gastric bypass, compared to 51% for sleeve gastrectomy. Similarly, the prevalence of olfactory changes was greater in the gastric bypass group (54%) compared to the sleeve gastrectomy group (46%). Lopes et al. [9] observed an increased frequency of olfactory changes alongside a pattern of significant initial and sustained long-term weight loss after bariatric surgery. This observation, consistent with the findings of Matsuo et al. [1], suggests a potential link between the extent of weight loss and alterations in olfactory function. 

Conversely, studies have documented a preoperative prevalence of olfactory dysfunction among individuals with severe obesity [4,10]. A study comparing patients with morbid obesity (body mass index, BMI 42.7 kg/m^2^) to normal controls (BMI 22.4 kg/m^2^) found an association between ghrelin levels (reduced in patients with morbid obesity) and smell impairment [10]. Therefore, for patients with obesity and preoperative olfactory dysfunction, bariatric surgery, in particular sleeve gastrectomy, has been shown to be effective in restoring olfactory decline [4].

In addition, several well-validated and reliable olfactory assessment tools are available [11]. Nevertheless, providing a more detailed rationale for selecting the olfactory visual analog scale in this study would enhance the reader’s understanding.

Lastly, the author stated that olfactory changes may be an independent factor in predicting weight loss following gastrectomy in patients with stomach cancer. However, a more comprehensive understanding of the underlying mechanisms, including the gut–brain axis, could lead to a reclassification of olfactory changes as a mediating factor. In this context, the mechanism itself might emerge as the independent cause of weight loss, making future research to elucidate this potential connection highly valuable.
